# Polymorphisms in *LRP2* and *CUBN* genes and their association with serum vitamin D levels and sleep apnea

**DOI:** 10.1007/s11325-023-02950-w

**Published:** 2023-11-27

**Authors:** Dimitra Anatolou, Paschalis Steiropoulos, Athanasios Zissimopoulos, Konstantina Chadia, Kostas Archontogeorgis, George Kolios, Vangelis G. Manolopoulos, Georgia Ragia

**Affiliations:** 1 Laboratory of Pharmacology, Medical School, Democritus University of Thrace, Dragana Campus, 68100 Alexandroupolis, Greece; 2 Individualised Medicine & Pharmacological Research Solutions Center (IMPReS), Alexandroupolis, Greece; 3 MSc Programme in Sleep Medicine, Medical School, Democritus University of Thrace, Dragana Campus, 68100 Alexandroupolis, Greece; 4 Department of Pneumonology, Medical School, Democritus University of Thrace, Dragana Campus, 68100 Alexandroupolis, Greece; 5 Laboratory of Nuclear Medicine, Medical School, Democritus University of Thrace, Dragana Campus, 68100 Alexandroupolis, Greece; 6 Clinical Pharmacology Unit, Academic General Hospital of Alexandroupolis, Alexandroupolis, Greece

**Keywords:** Obstructive sleep apnea syndrome, Vitamin D, Metabolism, *LRP2*, *CUBN*, Polymorphism

## Abstract

**Purpose:**

Vitamin D deficiency has been associated with the occurrence of obstructive sleep apnea syndrome (OSAS). Megalin (*LRP2*) and cubilin (*CUBN)* are implicated in vitamin D metabolism, whereas *LRP2* and *CUBN* polymorphisms have been previously associated with variable serum vitamin D levels. The present study aimed to evaluate the role of *LRP2* rs2228171 c.8614C > T and *CUBN* rs1801222 c.758A > G polymorphisms in OSAS susceptibility, independently or in synergy with vitamin D levels.

**Methods:**

Vitamin D serum concentration of consecutive individuals was measured. PCR–RFLP was used for *LRP2* rs2228171 and *CUBN* rs1801222 genotyping.

**Results:**

A total of 176 individuals was enrolled, including 144 patients with OSAS and 32 controls. Frequency of *LRP2* rs2228171 c.8614 T and *CUBN* rs1801222 c.758G alleles was estimated at 22.4% and 79.8%, respectively. *LRP2* and *CUBN* polymorphisms were not associated with OSAS occurrence (rs2228171Τ allele: 22.9% in OSAS group vs. 20.3% in controls, *p* = 0.651; rs1801222A allele 19.4% in OSAS group vs. 23.4% in controls, *p* = 0.471). Frequency of *CUBN* rs1801222A allele carriers was increased in patients with moderate or severe OSAS compared to mild OSAS (*p* = 0.028). Patients with OSAS homozygous for *LRP2* CC and *CUBN* GG genotypes had lower vitamin D serum concentration compared to controls carrying the same genotype (18.0 vs 27.0 ng/mL, *p* = 0.006 and 19.0 vs 27.5 ng/mL, *p* = 0.007, respectively).

**Conclusion:**

*CUBN* rs1801222 polymorphism may affect OSAS severity. Among other factors, low vitamin D concentration is associated with OSAS occurrence, irrespectively of *LRP2* and *CUBN* polymorphisms.

## Introduction

Obstructive sleep apnea syndrome (OSAS) is a multifactorial disorder leading to sleep disruption due to repetitive complete or partial upper airway obstructive events during sleep. OSAS diagnosis is based on sleep recordings; typical symptoms include loud snoring, daytime sleepiness, insomnia, fatigue, awakening with a choking or gasping sensation coupled with ≥ 5/h predominantly obstructive respiratory events, or a frequency of respiratory events ≥ 15/h (even in the absence of symptoms) [1]. OSAS is a quite common disorder affecting approximately 34% and 17% of men and women, respectively, with a variable frequency within ethnicities [2].

OSAS predisposing factors can be classified into different categories, including anatomical factors (such as retrognathia, laxity of the soft palate, macroglossia, large neck circumference), demographic factors (age, sex, race, family history), smoking, and alcohol consumption [3, 4], drugs that promote muscular relaxation and airway constriction (benzodiazepines, opiates), and endocrine disorders (hypothyroidism, polycystic ovarian syndrome) [5]. OSAS prevalence is increased in obese individuals with a range from 55 to 90%. Nevertheless, approximately 20% of patients with OSAS are non-obese, presenting, however, with less severe symptoms [4]. OSAS affects numerous comorbidities including stroke, diabetes, hyperlipidemia, depression, hypertension, and other cardiovascular diseases [5–8]. Additionally, in OSAS, the dysregulation of inflammatory biomarkers suggests a possible link between OSAS and inflammation [9]. Recent data suggest that low vitamin D levels and, thus, vitamin D deficiency are also associated with OSAS susceptibility [10–12]; however, the underlying molecular mechanisms are still unknown.

The two major forms of vitamin D are vitamin D2 (ergocalciferol) and vitamin D3 [13]. Vitamin D has a main role in calcium and phosphorus homeostasis regulation and in bone metabolism [14]. Calcitriol is an active metabolite of vitamin D prohormone, generated by a two-step metabolism performed in the liver and kidneys. Calcitriol regulates the expression of several genes binding to the vitamin D receptor (VDR) [15]. We have previously shown that gene polymorphisms in the *VDR* gene modulate vitamin D levels and are further associated with OSAS occurrence [16]. Therefore, seeking additional genetic markers within the vitamin D metabolic pathway may shed light on vitamin D–related OSAS molecular mechanism(s).

Two proteins that are implicated in the metabolic pathway of vitamin D, namely, megalin and cubilin, encoded by *LRP2* and *CUBN* genes, respectively, appear as attractive candidates for the genetic study of vitamin D variable concentration and their further implication in OSAS occurrence. Megalin and cubilin are endocytic receptors expressed in proximal tubule cells. They bind, among other ligands, with the vitamin D binding protein (VDBP) [17], the main transporter of 25-(OH) vitamin D3 [18] and mediate its uptake from glomerular filtrates. This process is essential for the release of the active form of vitamin D (1,25-(OH)2 vitamin D3) in the circulation [19].

Several polymorphisms have been identified in *LRP2* and *CUBN* genes that may contribute to modified vitamin D metabolism. Among them, *LRP2* rs2228171 (c.8614G > A) SNP (formerly assigned as rs4668123) leads to 2872Ala substitution with Thr, Ser, or Pro. This amino acid substitution is located within a megalin LDLa domain altering the polarity of the local site and affecting the structure of the protein, especially in the presence of a Pro residue. In Europeans, *LRP2* rs2228171 minor allele frequency (MAF) is approximately 27% [20]. This SNP has been previously associated with significantly higher vitamin D levels (p-adj 0.007) [21]. *CUBN* rs1801222 (c.758 T > C) SNP is a missense variant leading to 253Phe substitution with Ser or Cys, affecting, thus, cubilin polarity and hydrophobic nature. Rs1801222 MAF in European populations is as high as 67% [20]. A previous study showed a tendency for higher levels of vitamin D in C carriers, albeit differences were nonsignificant [21].

The aim of the present study was to assess the association of the *LRP2* rs2228171 c.8614G > A and *CUBN* rs1801222 c.758 T > C gene polymorphisms with the emergence of OSAS and OSAS severity either independently or in interaction with vitamin D deficiency.

## Subjects and methods

### Patients

**Fig. 1:**
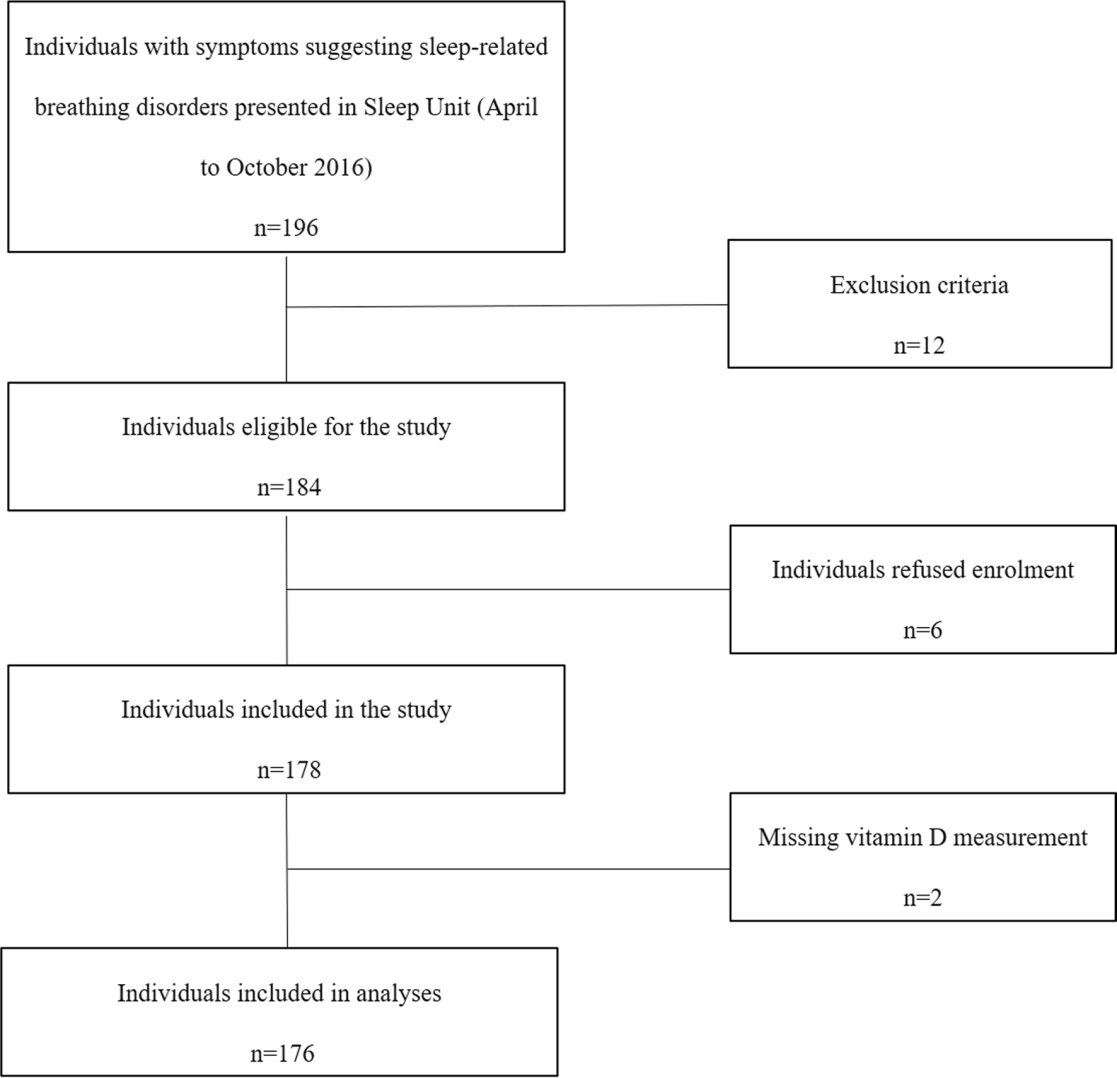
Analytic flowchart of selection of individuals included in the study

The patient group comprised consecutive patients in our institution’s sleep unit, whose symptoms suggest respiratory issues related to sleep. Cohort characteristics, exclusion criteria, sample collection, and preparation processes, as well as apnea and hypopnea definitions according to apnea–hypopnea index (AHI) and vitamin D concentration grading have been described in detail in our previous study [16]. An analytic flowchart of individuals included in the study is presented in Fig. 1. The study was carried out in accordance with the Helsinki Declaration of Human Rights, and patients gave their informed consent [22]. The study protocol was approved by the Scientific Council and the Ethics Committee of Academic General Hospital of Alexandroupolis, Greece (protocol approval 33/19–12-2014).

### Genotyping

The genomic DNA extraction process has been described in detail in our previous study [16]. Quawell Q5000 UV–Vis Spectrophotometer (Quawell Technology, Inc., San Jose, CA, USA) was used to determine DNA concentration and purity. Polymerase chain reaction and restriction fragment length polymorphism (PCR–RFLP) analysis was used for the identification of *LRP2* rs2228171 c.8614G > A and *CUBN* rs1801222 c.758 T > C polymorphisms, as described elsewhere [21], with identical PCR conditions except for an annealing temperature of 57 °C for both PCR reactions, using *Fsp*I (NIPPON Genetics EUROPE, Düren, Germany) and *Bbs*I (New England BioLabs, Hitchin, UK) restriction enzymes, respectively. The opposite strand was genotyped for the detection of both polymorphisms, and thus variant alleles are assigned throughout the manuscript as *LRP2* rs2228171C > T and *CUBN* rs1801222A > G. All PCR–RFLP procedures were carried out in Mastercycler® nexus PCR Thermal Cycler (Eppendorf SE, Hamburg, Germany).

### Statistical analyses

IBM SPSS Statistics for Windows, Version 20.0 (Armonk, NY: IBM Corp.) was used for statistical analyses. Expression of continuous and non-continuous variables and all statistical analyses regarding them have been described previously in detail [16]. The same tests and factors were used for the estimation of the deviation from the Hardy–Weinberg equilibrium (HWE) and the odds ratio (OR) in order to evaluate the risk of OSAS associated with each polymorphism, as well as the vitamin D concentration. Differences resulting from the comparisons were considered statistically significant at a *p* value < 0.025 after Bonferroni correction.

## Results

### Population characteristics

Demographic and clinical characteristics of patients with OSAS and healthy controls are shown in Table 1 and have been extensively described in our previous work, combined with other differences in anthropometric and sleep parameters [16].

**Table 1:** Demographic, anthropometric, and biochemical characteristics of OSAS patients and controls

	OSAS patients (*n* = 144)	Controls (*n* = 32)	*P* value
Male, n (%)	122 (84.7)	19 (59.4)	0.001
Age, years (mean ± SD)	53.2 ± 12.4	47.6 ± 14.3	0.025
BMI, kg/m^2^ (mean ± SD)	35.4 ± 6.9	30.3 ± 6.4	< 0.001
Vitamin D concentration, ng/ml (median, 25, 75 percentiles)	19.0 (12.0, 27.0)	27.0 (16.5, 32.0)	0.006
Total cholesterol, mg/dl (mean ± SD)	206.1 ± 42.6	205.4 ± 41.0	NS
LDL-cholesterol, mg/dl (mean ± SD)	123.4 ± 36.1	120.8 ± 37.8	NS
HDl-cholesterol, mg/dl (median, 25, 75 percentiles)	45.0 (41.0, 52.5)	44.0 (36.0, 58.0)	NS
Triglycerides, mg/dl (median, 25, 75 percentiles)	146.0 (103.5, 194.5)	149.5 (83.3, 227.8)	NS
Glucose, mg/dl (median, 25, 75 percentiles)	100.0 (90.5, 117.5)	96.5 (81.5, 106.3)	NS
Circumference, cm			
Neck (mean ± SD)	44.3 ± 3.9	39.6 ± 3.8	< 0.001
Waist (median, 25, 75 percentiles)	121.0 (111.5, 129.5)	100.0 (96.0, 118.0)	< 0.001
Hip (median, 25, 75 percentiles)	117.0 (110.0, 124.0)	1090 (105.0, 116.0)	0.006

*OSAS* obstructive sleep apnea syndrome, *BMI* body mass index, *NS* nonsignificant, *LDL* low density lipoprotein, *HDL* high density lipoprotein

### *LRP2* and *CUBN* polymorphisms distribution in OSAS patients and controls

In the total cohort, the frequency of the *LRP2* rs2228171 T and *CUBN* rs1801222 G alleles was estimated at 22.4% and 79.8%, respectively. Genotypes for both genes were in Hardy–Weinberg equilibrium (*LRP2*; χ^2^ 0.24, *p* = 0.62, *CUBN*; χ^2^ 0.006, *p* = 0.94). Genotype and allele distribution for both polymorphisms did not differ between the OSAS group and controls (Table 2). Also, there was no association with OSAS for none of the polymorphisms when the dominant or recessive model of inheritance was applied (data not shown). In terms of OSAS severity, *CUBN* rs1801222 A allele carriage was more common as moving from mild to moderate and severe OSAS (*p* = 0.028) (Table 3). No other associations were found.

**Table 2:** Frequency of *LRP2* and *CUBN* genotypes and alleles in total cohort and stratified as OSAS patients and controls

	Total cohort (*n* = 176)		OSAS (*n* = 144)		Controls (*n* = 32)		*p* Value
	*n* (%)	95% CI	*n* (%)	95% CI	*n* (%)	95% CI	
*LRP2*							
CC	107 (60.8)	53.5–67.8	88 (61.1)	52.9–68.8	19 (59.4)	42.2–74.9	0.244
CT	59 (33.5)	26.9–40.7	46 (32.0)	24.7–39.9	13 (40.6)	25.0–57.8	
TT	10 (5.7)	2.9–9.8	10 (6.9)	3.6–11.9	0	-	
C allele	273 (77.6)	72.9–81.7	222 (77.1)	71.9–81.6	51 (79.7)	68.6–88.1	0.651
T allele	79 (22.4)	18.3–27.0	66 (22.9)	18.4–28.0	13 (20.3)	11.9–31.4	
*CUBN*							
GG	112 (63.6)	56.3–70.5	94 (65.3)	57.3–72.7	18 (56.3)	39.2–72.3	0.542
GA	57 (32.4)	25.8–39.5	44 (30.5)	23.5–38.4	13 (40.6)	25.0–57.8	
AA	7 (4.0)	1.8–7.7	6 (4.2)	1.8–8.4	1 (3.1)	0.3–13.7	
G allele	281 (79.8)	75.4–83.8	232 (80.6)	75.7–84.8	49 (76.6)	65.2–85.6	0.471
A allele	71 (20.2)	16.2–24.6	56 (19.4)	15.2–24.3	15 (23.4)	14.4–34.8	

*OSAS* obstructive sleep apnea syndrome, *LRP2* megalin, *CUBN* cubilin

**Table 3:** Frequency of *LRP2* and *CUBN* genotypes and alleles in patients according to OSAS severity

	OSAS severity			
	Mild (*n* = 29)	Moderate (*n* = 14)	Severe (*n* = 101)	*p* Value
*LRP2*				
CC	18 (62.1)	10 (71.4)	60 (59.4)	0.826
CT	10 (34.5)	3 (21.4)	33 (32.7)	
TT	1 (3.4)	1 (7.1)	8 (7.9)	
T allele carriers	11 (37.9)	4 (28.6)	41 (40.6)	0.683
*CUBN*				
GG	25 (86.2)	9 (64.3)	60 (59.4)	0.104
GA	4 (13.8)	4 (28.6)	36 (35.6)	
AA	0	1 (7.1)	5 (5.0)	
A allele carriers	4 (13.8)	5 (35.7)	41 (40.6)	0.028

*OSAS* obstructive sleep apnea syndrome, *LRP2* megalin, *CUBN* cubilin

### Effect of *LRP2* and *CUBN* polymorphisms on vitamin D serum concentration

Participants were classified as having normal levels of vitamin D (20 ng/ml) or vitamin D deficiency (< 20 ng/ml). Between two groups, no differences were observed in *LRP2* and *CUBN* genotype and allele distribution (Table 4). When vitamin D was treated as a continuous variable, *LRP2* and *CUBN* genotypes did not affect vitamin D concentration in total cohort. Since vitamin D shows in our population a high association with OSAS occurrence [16], the same analysis was performed in participants stratified as OSAS patients and controls. We have observed a trend, albeit non-significant, towards higher vitamin D levels for each *LRP2* T allele carriage both in patients and controls, whereas for *CUBN* G allele, the same trend was present in control group (Table 5). No association of *LRP2* and *CUBN* genotypes with vitamin D levels was present when dominant and recessive model of inheritance were applied (data not shown). Within distinct genotype analysis, patients with OSAS carrying the *LRP2* CC genotype and *CUBN* GG genotypes had significantly lower vitamin D concentration compared to *LRP2* CC and *CUBN* GG controls (18.0 vs 27.0 ng/mL, *p* = 0.006 and 19.0 vs 27.5 ng/mL, *p* = 0.007, respectively) (Table 5).

**Table 4:** Frequency of *LRP2* and *CUBN* genotypes and alleles in vitamin D deficient and normal cases

	Vitamin D deficient (< 20 ng/mL, *n* = 84)		Vitamin D normal (≥ 20 ng/mL, *n* = 92)		*p* Value
	*n* (%)	95% CI	*n* (%)	95%CI	
*LRP2*					
CC	51 (60.7)	50.0–70.7	56 (60.9)	50.7–70.4	0.866
CT	29 (34.5)	25.0–45.1	30 (32.6)	23.7–42.6	
TT	4 (4.8)	1.6–10.9	6 (6.5)	2.8–12.9	
C allele	131 (78.0)	71.3–83.7	142 (77.2)	70.2–82.8	0.857
T allele	37 (22.0)	16.3–28.7	42 (22.8)	17.2–29.3	
*CUBN*					
GG	52 (61.9)	51.3–71.7	60 (65.2)	55.1–74.4	0.832
GA	28 (33.3)	23.9–43.8	29 (31.5)	22.7–41.5	
AA	4 (4.8)	1.6–10.9	3 (3.3)	0.9–8.5	
G allele	132 (78.6)	71.9–84.3	149 (81.0)	74.8–86.1	0.574
A allele	36 (21.4)	15.7–28.1	35 (19.0)	13.9–25.2	

*OSAS* obstructive sleep apnea syndrome, *LRP2* megalin, *CUBN* cubilin

**Table 5:** Vitamin D levels in total cohort, patients, and controls according to their *LRP2* and *CUBN* genotypes

	Total cohort (*n* = 176)		OSAS (*n* = 144)		Controls (*n* = 32)		*p Value
	Vitamin D	*p* Value	Vitamin D	*p* Value	Vitamin D	*p* Value	
*LRP2*							
CC	20.0 (13.0, 27.0)	0.709	18.0 (12.0, 26.0)*	0.466	27.0 (19.0, 32.0)*	0.591	0.006
CT	20.0 (12.0, 30.0)		18.5 (9.8, 30.5)		28.0 (14.5, 31.5)		
TT	22.5 (18.8, 27.5)		22.5 (18.8, 27.5)		-		
*CUBN*							
GG	21.0 (13.0, 29.75)	0.658	19.0 (12.8, 27.0)*	0.723	27.5 (19.8, 33.8)*	0.188	0.007
GA	20.0 (12.0, 28.0)		18.0 (10.3, 26.0)		25.0 (17.0, 30.0)		
AA	18.0 (11.0, 31.0)		19.0 (14.5, 32.5)		19.0		

*OSAS* obstructive sleep apnea syndrome, *LRP2* megalin, *CUBN* cubilin

### Regression analysis for OSAS risk and vitamin D concentration

To evaluate the risk of OSAS associated with *LRP2* and *CUBN* gene polymorphisms, logistic regression analysis was performed, adjusted for other factors related to OSAS (age, gender, BMI, vitamin D levels, comorbidities, smoking, sleep efficiency). The following variables were associated with OSAS occurrence: age (OR = 1.075, 95% CI = 1.020–1.133, *p* = 0.007), gender (OR = 0.059, 95% CI = 0.014–0.248, *p* < 0.001), BMI (OR = 1.234, 95% CI = 1.103–1.381, *p* < 0.001), vitamin D (OR = 0.942, 95% CI = 0.907–0.979, *p* = 0.002), and sleep efficiency (OR = 1.062, 95% CI = 1.017–1.109, *p* = 0.006), whereas no association of *LRP2* and *CUBN* polymorphisms was found.

Linear regression analysis with vitamin D as the dependent variable and age, gender, BMI, comorbidities, smoking, and *LRP2* and *CUBN* gene polymorphisms as independent variables showed that none of the polymorphisms is associated with vitamin D serum concentration. When the term interaction was inserted in the model, the interaction of vitamin D with *LRP2* polymorphism was significantly associated with the OSAS risk (OR = 0.978, 95% CI = 0.960–0.996, *p* = 0.019) and, additionally, with age (OR = 1.068, 95% CI = 1.016–1.123, *p* = 0.009), gender (OR = 0.063, 95% CI = 0.017–0.239, *p* < 0.001), BMI (OR = 1.223, 95% CI = 1.099–1.362, *p* < 0.001), and sleep efficiency (OR = 1.058, 95% CI = 1.013–1.105, p = 0.011). Similarly, the interaction of vitamin D with *CUBN* polymorphism was significantly associated with the OSAS risk (OR = 0.970, 95% CI = 0.950–0.990, *p* = 0.004), with age (OR = 1.069, 95% CI = 1.017–1.124, *p* = 0.008), gender (OR = 0.071, 95% CI = 0.019–0.263, *p* < 0.001), BMI (OR = 1.225, 95% CI = 1.101–1.363, *p* < 0.001), and sleep efficiency (OR = 1.054, 95% CI = 1.011–1.098, *p* = 0.013).

## Discussion

The present study assessed the effect of *LRP2* rs2228171 c.8614C > T and *CUBN* rs1801222 c.758A > G gene polymorphisms on vitamin D concentration and on OSAS occurrence, independently or in interaction with vitamin D levels. This is the first study examining the potential implication of *LRP2* and *CUBN* gene polymorphisms in OSAS occurrence, in association with vitamin D deficiency. We have shown that *LRP2* CC and *CUBN* GG genotypes were associated with significantly lower vitamin D concentration in patients with OSAS compared to healthy controls. Additionally, *CUBN* A allele carriage frequency was increased in moderate or severe OSAS compared to the frequency in patients with mild symptoms.

Different polymorphisms of *LRP2* and *CUBN* genes have been previously associated with variable vitamin D concentration [23–26]. More specifically, *LRP2* rs2673170 and rs10210408 and *CUBN* rs4525114 polymorphisms have been associated with season-adjusted 25(OH)D concentration among controls or pregnant women [25, 27]. Additionally, *LRP2* rs2075252 variant has been associated with vitamin D deficiency [24], while *CUBN* rs41301097 has been strongly correlated with higher 25(OH)D levels [19]. *LRP2* rs2228171 and *CUBN* rs1801222 polymorphisms, that were genotyped in our study, have been previously associated with variable vitamin D levels [21]; our results in total population did not show such an association. In control group, however, we observed a similar trend towards increased vitamin D levels in *LRP2* T and *CUBN* G carriers.

Previous studies have shown that vitamin D serum concentration is lower in patients with OSAS [10, 11, 16, 28] and this reduction is more pronounced as the severity of the disease increases [29]. Vitamin D should be activated via metabolism in order to exert its biological functions [15]. Several genes are implicated in vitamin D metabolic pathway. Therefore, gene polymorphisms possibly affecting this pathway appear as attractive candidates to study the genetic architecture of vitamin D deficiency and OSAS genetics. The uptake of the complex 25-(OH)D3-VDBP by the kidneys is performed by *LRP2*- and *CUBN*-mediated endocytosis [18, 30]. This process produces the active form of the vitamin and maintains its serum concentration [30]. Therefore, functional alterations in *LRP2* and *CUBN* genes can potentially result in increased urinary concentration of the 25-(OH)D3-VDBP complex and, subsequently, in reduced activation of 1,25-(OH)2 vitamin D3 [31]. However, since the functional effect of the above polymorphisms has not been fully elucidated, our results can only suggest a minor role of *LRP2* and *CUBN* gene polymorphisms in sleep apnea possibly mediated via vitamin D levels.

Additionally, our study showed that the vitamin D levels are significantly lower in patients with OSAS with *LRP2* CC and *CUBN* GG genotypes than in healthy homozygous controls. Additionally, *CUBN* A allele carriage that was hypothesized to be associated with decreased vitamin D levels was increased in frequency in patients with moderate or severe OSAS symptoms. To date, there are no other studies on the potential role of *LRP2* rs2228171 c.8614C > T and *CUBN* rs1801222 c.758A > G gene polymorphisms in OSAS. To further evaluate whether the specific variations or the vitamin D levels have a more prominent effect on OSAS, we performed two different regression models, including other OSAS risk factors. We found that vitamin D concentration alone and its interaction with the above polymorphisms are associated with OSAS occurrence (for *LRP2*, OR = 0.978, 95% CI = 0.960–0.996, *p* = 0.019, and for *CUBN*, OR = 0.970, 95% CI = 0.950–0.990, *p* = 0.004). However, since the polymorphisms are not independently associated with OSAS, these results should be interpreted with caution and can be attributed to the strong association of vitamin D concentration per se with OSAS in our population.

Our study has several strengths as they have been previously described [16]. It should be acknowledged, however, herein that several limitations also exist. Levels of vitamin D both in patients and controls were not continuously measured. Additionally, studied SNPs were selected based on their potential functionality and association with levels of serum vitamin D and their MAF; it cannot be excluded that other gene variants may have a more pronounced effect on protein function. In our study, patients with OSAS and controls were not age or gender matched. Therefore, larger studies are necessary to replicate the results on the role of *LRP2* and *CUBN* gene polymorphisms in vitamin D serum concentration in OSAS.

In conclusion, the present study aimed to evaluate the role of the *LRP2* rs2228171 c.8614G > A and *CUBN* rs1801222 c.758 T > C gene polymorphisms in the serum vitamin D concentration and their association with the occurrence of OSAS and OSAS severity. The results showed that the studied polymorphisms do not independently affect OSAS occurrence; however, OSAS severity may be affected by *CUBN* genetic variation. Differences in vitamin D levels between patients and controls are noticed within distinct *LRP2* and *CUBN* genotypes suggesting an underlying role of the studied genes in vitamin D metabolism. Vitamin D-related mechanisms that may contribute to OSAS risk should be explored in greater depth.

## Data Availability

The datasets generated during and/or analyzed during the current study are available from the corresponding author on reasonable request.

## Declarations

Approval was obtained from the Scientific Council and the Ethics Committee of Academic General Hospital of Alexandroupolis, Greece (protocol approval 33/19–12-2014). The procedures used in this study adhere to the tenets of the Helsinki Declaration of Human Rights. Informed consent was obtained from all individual participants included in the study.
